# Level of patient contact and Impact of Event scores among Canadian healthcare providers during the COVID-19 pandemic

**DOI:** 10.1186/s12913-024-11426-w

**Published:** 2024-08-20

**Authors:** Iris Gutmanis, Ayodele Sanni, Allison McGeer, Robert Maunder, Nicole Robertson, CCS Working Group, Curtis Cooper, Kevin Katz, Mark Loeb, Shelly McNeil, Matthew Muller, Jeff Powis, Robyn Harrison, Joanne Langley, Samira Mubareka, Jeya Nadarajah, Louis Valiquette, Marek Smieja, Brenda L Coleman

**Affiliations:** 1 Sinai Health System, 600 University Ave, Toronto, ON M5G 1X5 Canada; 2 University of Toronto, 27 King’s College Circle, Toronto, ON, M5S 1A1 Canada

**Keywords:** Post-traumatic stress, Healthcare provider, COVID-19, IES-R

## Abstract

**Background:**

Healthcare providers (HCP) continue to provide patient care during the COVID-19 pandemic despite the known risks for transmission. Studies conducted early in the pandemic showed that factors associated with higher levels of distress among HCP included being of younger age, female, in close contact with people with COVID-19, and lower levels of education. The goal of this study was to determine if level of patient contact was associated with concern for post-traumatic stress disorder (PTSD) as measured by the Impact of Event Scale-Revised (IES-R).

**Methods:**

This cross-sectional study, embedded within a prospective cohort study, recruited HCP working in hospitals in four Canadian provinces from June 2020 to June 2023. Data were collected at enrolment and annually from baseline surveys with the IES-R scale completed at withdrawal/study completion. Modified Poisson regression was used to determine the association between level of patient contact and concern for PTSD (i.e., IES-R scores ≥24).

**Results:**

The adjusted rate ratio (RR) associated with concern for PTSD among HCP with physical contact/direct patient care was 1.19 (95% confidence interval (CI) 1.03, 1.38) times higher than for HCP with no direct contact. In fully adjusted linear regression models, physical care/contact was associated with higher avoidance and hyperarousal scores, but not intrusion scores.

**Conclusions:**

Administrators and planners need to consider the impact of heightened and ongoing stress among HCP by providing early screening for adverse emotional outcomes and delivery of tailored preventive strategies to ensure immediate and long-term HCP health.

**Supplementary Information:**

The online version contains supplementary material available at 10.1186/s12913-024-11426-w.

## Background

Transmission of the SARS-CoV-2 coronavirus has continued since the World Health Organization (WHO) declared coronavirus disease 2019 (COVID-19) a pandemic on March 11, 2020 [1]. Due to the significant physical health impacts of COVID-19, attempts were made to slow viral transmission including quarantine restrictions and preventive methods such as wearing masks, social distancing, closing venues, and vaccination. However, such mitigation strategies, added to the threats of the disease itself, can increase disruption and trauma-related stress [2, 3].

Post-traumatic stress disorder (PTSD) is a common outcome after witnessing or experiencing a traumatic event [4]. In a 2002 Canadian study based on self-reported symptoms, Van Ameringen et al. found that prevalence of PTSD was 2.4% [5]. In a subsequent self-reported Canadian survey conducted between August and December 2021, the estimated prevalence of PTSD had increased to 5.4% [6]. Despite ongoing transmission, healthcare providers (HCP) need to provide optimal patient care. During the early pandemic period higher stress levels were reported by HCP secondary to increased workload, constantly changing work environments, provision of care to people with COVID-19, deaths caused by the disease, and fear of infecting themselves and their close contacts [7, 8].

Several studies [9–15] have used the Impact of Event Scale-Revised (IES-R) [16] to assess concern for PTSD during the COVID-19 pandemic. Among HCP, younger age, female gender, personal exposure to COVID-19, and lower levels of education have been identified as factors associated with higher levels of concern for PTSD [11, 14, 17]. However, these studies were conducted over short periods and/or early in the pandemic when some transmission mitigation strategies, such as quarantine measures and vaccines, had either not been employed or were not yet available.

This study aims to investigate the level of concern for PTSD, as measured by the IES-R, among Canadian HCP in relation to their level of contact with patients, adjusted for potential confounders, between June 10, 2021 and December 1, 2023. Levels of avoidance, intrusion, and hyperarousal are also explored.

## Methods

### Study design

The COVID-19 Cohort Study was a 3.5 year prospective cohort study following HCP from acute care, rehabilitation, and complex care hospitals in the greater Toronto area (Sinai Health, Sunnybrook Health Sciences Centre, Oak Valley Health (Markham Stouffville Hospital), North York General, Michael Garron Hospital, Unity Health (St. Michael’s Hospital), William Osler Health System, and University Health Network), Hamilton (St. Joseph’s Healthcare Hamilton, Hamilton Health Sciences Centre), Ottawa (The Ottawa Hospital), Alberta (Calgary Health Zone, Grey Nuns Community Hospital, University of Alberta Hospital), Quebec (Centre hospitalier universitaire de Sherbrooke), and Halifax (IWK Health Centre, QE II) as well as private physician or midwifery practices in the Toronto area. Rolling enrollment, from June 2020 to June 2023, occurred following ethical approval at each site.

**Fig. 1:**
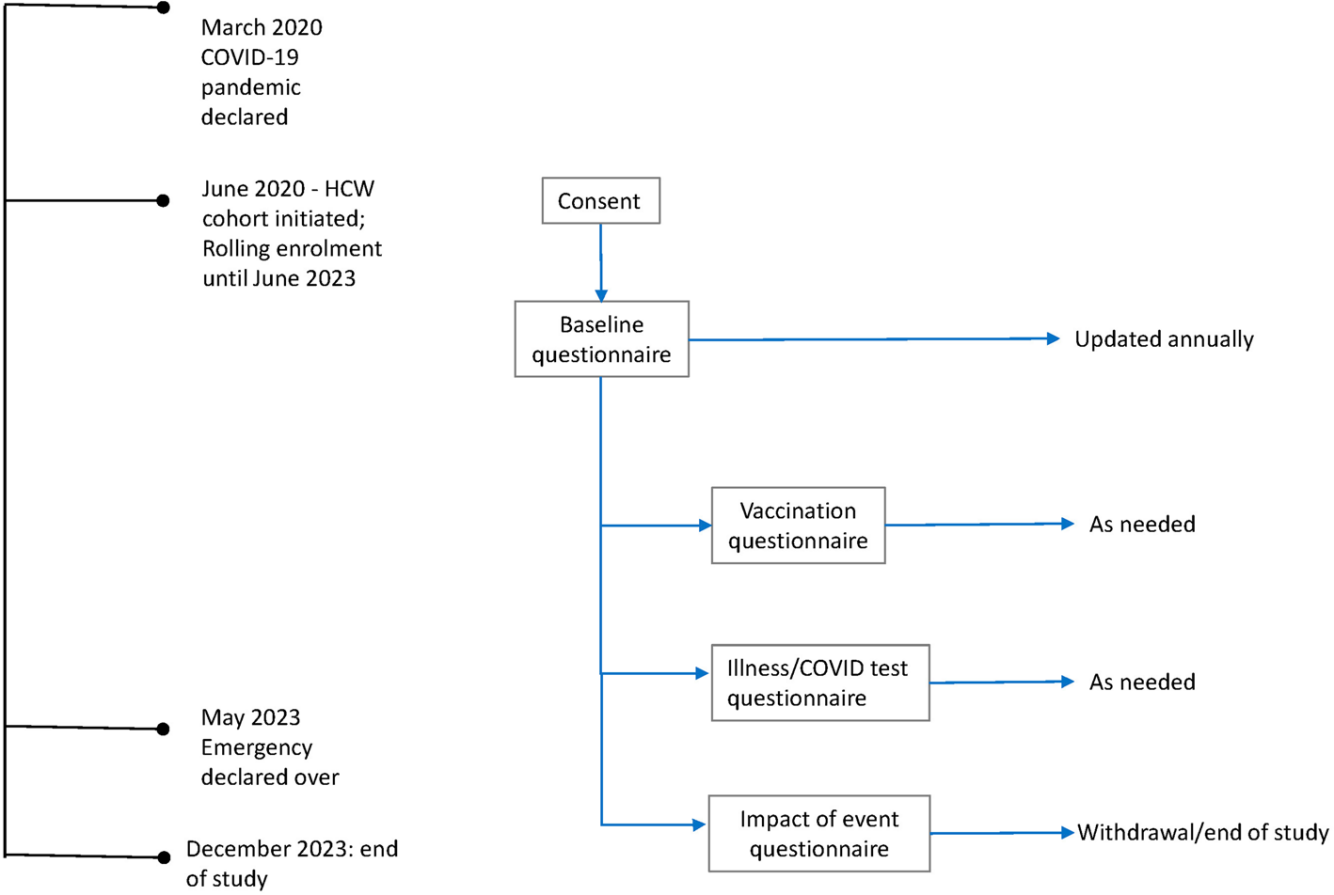
Study design and timeline

Consented participants completed annual baseline surveys and illness and vaccination surveys as needed. Participants completed the IES-R once within two weeks of study withdrawal or at study closure (December 1, 2023) (see Fig. 1).

### Participants

Consented HCP were eligible for these analyses if they completed ≥ 50% of their most recent baseline questionnaire; were 18 to 75 years old, inclusive; were employed full- or part-time (> 20 h per week) by a participating hospital; or were a physician, midwife, or nurse practitioner with hospital privileges and who cared for ill patients ≥ 8 h per week. Participants with incomplete IES-R data were excluded from analyses.

### Outcome

The Impact of Event Scale (IES) is a 15-item measure of the frequency with which respondents experience thoughts and behaviours associated with two definitional symptoms of PTSD: intrusion and avoidance [18]. The scale was revised (IES-R) with the addition of another set of items designed to identify the frequency of hyperarousal symptoms [16]. The IES-R asks participants to indicate, on a scale from 0 (not at all) to 4 (extremely), how distressing each of 22 listed difficulties have been for them during the previous seven days. To orient participants, the survey was introduced with “You have been working throughout the COVID-19 pandemic”. Overall IES-R scores are the sum of all 22 items (range 0 to 88) and were interpreted using criteria developed by Weiss and Marmar [16] (0–23: no concern for PTSD; ≥ 24: indicative of concern for PTSD). Subscale scores (avoidance, intrusion, hyperarousal) are the mean of the subscale item scores (range 0 to 4).

### Exposure

In the baseline survey, participants were asked to respond to three questions that asked them to indicate which of five levels of contact they had with inpatients, outpatients, and emergency department patients: 1) not applicable, no close contact with patients; 2) never or rarely in room or confined physical space with patients; 3) in room/confined space with patients, but not within 2 m of their face; 4) in room/confined space and within 2 m of their face, but no physical contact/care; 5) physical contact and/or care of patients. Two metres was selected as a key distance for this study as recommended by the Government of Canada to reduce transmission of COVID-19 [19]. The highest level of contact in any of the three settings was selected. The four levels of patient contact used in these analyses are: 1) no direct contact; 2) never/rarely in patient rooms; 3) in patient rooms but no contact, and 4) physical care/contact.

### Covariates

Baseline surveys collected demographic and work-related information including age; gender; self-reported health; medications for anxiety, depression, or insomnia; occupation; and current work unit (high-risk: emergency department, adult intensive care unit, or adult inpatient medical unit vs low-risk: all others). COVID-19 vaccine receipt was obtained from the most recent vaccination questionnaire. The study did not use information from the illness/testing surveys.

A mitigation strategy measure that ranked the intensity of non-pharmacological mitigation strategies associated with three sectors (work, education, and other locations) on a four-point scale (0: no restrictions to 3: most stringent restrictions) was included as a study covariate [20]. The mutually exclusive categories developed by Akanteva et al. are specific to each sector (e.g., category 3: work: all non-essential workplaces closed or operating remotely, only essential services or businesses remain open; education: all schools closed for in-person instruction; other locations: stringent gathering restriction, border closures between provinces for non-essential travel, closure of all indoor activities, and closure of most outdoor activities) [20]. Periods when restrictions summed to  ≥7  were identified as periods of high mitigation. Periods were determined using Ontario data. While other provinces imposed different restrictions at different times, Ontario was selected as the referent as most study sites were within this province.

### Data analysis

Chi-squared or Fisher’s exact tests were used to compare categorical variables while t-tests or median tests were used to compared continuous variables, as appropriate. Modified Poisson regression was used to quantify the relationships between study variables and dichotomized IES-R scores [21]. All models were adjusted by age and gender. Other study covariates that were not associated with the outcome were sequentially eliminated as per Vittinghoff et al. [22]. Variance estimates were adjusted in multivariable models for clustering within province. The final model was assessed for goodness-of-fit and multi-collinearity. Linear regression, adjusted for clustering within provinces, was used to assess the association between subscale scores and variables found to be significant predictors of the dichotomized IES-R score.

## Results

### Participant characteristics

Of the 2648 HCP who participated in the parent study, 1498 (56.6%) submitted an IES-R between June 10, 2021 and December 1, 2023. Those who submitted an IES-R were significantly older, and less likely to be a nurse, work on a high-risk unit, or to have been working for less than five years compared with the full cohort (see Table 1).

**Table 1:** Canadian healthcare provider characteristics of participation: COVID-19 Cohort Study (June 15, 2020-December 1, 2023) versus IES-R sub-study (June 10, 2021-December 1, 2023)

**Variable**	Full cohort study (*N*=2648)	IES-R sub-study (*N*=1498)	*p*-value^1^
*Gender:* Female	2279 (86.1)	1308 (87.3)	0.05
Male	360 (13.6)	190 (12.7)	
Other	9 (0.3)	0 (0.0)	
*Age at enrolment*, mean (95% CI)	41.1 (40.7, 41.6)	42.4 (41.9, 43.0)	<0.001
*Education, highest completed*			0.79
College diploma or less	667 (25.2)	369 (24.6)	
Bachelor's degree	1149 (43.4)	650 (43.4)	
Master's degree	468 (17.7)	278 (18.6)	
MD/PhD	362 (13.7)	201 (13.4)	
Missing	2 (0.1)	0	
*Health status, self-reported*			0.77
Poor/fair/good	610 (23.1)	343 (22.9)	
Very good	1250 (47.2)	723 (48.3)	
Excellent	788 (29.8)	432 (28.8)	
*COVID-19 vaccination status*			NA^3^
Incomplete		154 (10.3)	
Fully vaccinated^2^		1344 (89.7)	
*Works on a high-risk unit* ^4^			<0.001
No	1777 (67.1)	1060 (70.8)	
Yes	837 (31.6)	434 (29.0)	
Missing	34 (1.3)	4 (0.3)	
*Occupation*			0.003
Nurse (NP/RN/RPN)/midwife	893 (33.7)	482 (32.2)	
Physician/physician assistant	283 (10.7)	152 (10.1)	
Other regulated HCP^5^	764 (28.9)	463 (30.9)	
Other^6^	684 (25.8)	401 (26.8)	
Missing	24 (0.9)	0	
*Province of work*			0.72
Ontario	1595 (60.2)	875 (58.4)	
Alberta	486 (18.4)	285 (19.0)	
Quebec	324 (12.2)	194 (13.0)	
Nova Scotia	243 (9.2)	144 (9.6)	
*Highest level of patient contact*			<0.001
No patient care	470 (17.7)	300 (20.0)	
Never/rarely in room	233 (8.8)	145 (9.7)	
Same room	489 (18.5)	280 (18.7)	
Physical care/contact	1430 (54.0)	773 (51.6)	
Missing	26 (1.0)	0	

*HCP* healthcare provider, *NP* Nurse practitioner, *RN* Registered nurse, *RPN* Registered practical nurse, *NA* Not applicable

^1^Pearson’s chi-squared for binary/categorical variables, two-sided t-test for continuous variables

^2^See definition [23]

^3^No appropriate comparison available for the full-study population given that COVID-19 vaccination status was a time-varying factor

^4^Adult intensive care units, emergency departments, and adult inpatient medical units

^5^Respiratory therapist, laboratory technician, physical therapist, occupational therapist, imaging technician/technologist, pharmacist, pharmacy technician, psychologist, social worker

^6^Infection prevention and control practitioner, food service, ward clerk, administration, healthcare aid, housekeeper, porter, researcher, other clinical support

Almost one third of participants who completed the IES-R were nurses, nurse practitioners, or midwives, 773 (51.6%) provided physical care to patients, and 875 (58.4%) worked in the province of Ontario. The majority of participants were female (1309 or 87.3%) and had a mean age of 42.4 (95% confidence interval (CI) 41.9, 43.0) years. As gender was considered an important study covariate, those who selected “other” were dropped from analyses due to the small sample size.

The top three individual IES-R items most frequently assigned a score of 4, indicating that participants were extremely affected, were trouble staying asleep (*n* = 48/1498 or 3.2%), trouble falling asleep (*n* = 42), and trying not to think about it (*n* = 39). Meanwhile, few (*n* = 6) indicated they were extremely affected by reminders causing physical reactions, such as sweating, trouble breathing, nausea, or a pounding heart.

As shown in Table 2, median IES-R scores, mean subscale scores, and the percent of respondents with an IES-R score indicative of concern for PTSD were similar in 2021 and 2022 but decreased significantly in 2023 (*p* < 0.001).

**Table 2:** Crude IES-R and subscale scores for Canadian healthcare providers, June 10, 2021-December 1, 2023, by calendar year

Survey tool	2021 (Jun. 10 to Dec. 31) (*n* = 268)	2022 (Jan. 1 to Dec. 31) (*n* = 928)	2023 (Jan. 1 to Dec. 1) (*n* = 302)	Overall (*n* = 1498)
Total IES-R^a^	22 (9, 35)	21 (7, 34)	10 (2, 22)	18 (6, 32)
IES-R score ≥ 24^b^	128 (47.8%)	416 (44.8%)	68 (22.5%)	612 (40.8%)
Avoidance^c^	1.00 (0.25, 1.62)	1.00 (0.25, 1.75)	0.37 (0, 1.12)	0.87 (0.12, 1.62)
Intrusion^c^	1.12 (0.25, 1.75)	0.87 (0.31, 1.62)	0.50 (0.12, 1.12)	0.87 (0.25, 1.50)
Hyperarousal^c^	0.83 (0.33, 1.50)	0.83 (0.17, 1.50)	0.33 (0, 0.83)	0.67 (0.17, 1.33)

Median test for IES-R scores over time: *p* < 0.001

^a^median (interquartile range) as IES-R scores were not normally distributed

^b^number (percent)

^c^mean (95% confidence interval)

### Patient contact and concern for PTSD

As seen in Table 3, the unadjusted rate ratio (RR) associated with concern for PTSD among HCP with physical contact/care was 1.24 (95% CI 1.05 1.47) times higher than for HCP with no direct contact. When confounding variables were added, the adjusted RR associated with physical contact/care decreased by less than 5% to 1.19 (95% CI 1.03, 1.38).

**Table 3:** Factor-specific and modified Poisson regression model estimates comparing IES-R scores of no concern (<24) versus of concern (≥24), Canadian healthcare providers (June 10, 2021-December 1, 2023)

	**Factor-specific estimates** (*N*=1498) RR (95% CI)	Adjusted model^a^ (*N*=1498) RR (95% CI)
*Level of patient contact*		
No direct contact	Referent	Referent
Never/rarely in room	0.99 (0.76, 1.28)	0.96 (0.76, 1.20)
Same room	1.16 (0.95, 1.41)	1.12 (0.99, 1.28)
Physical contact/care	1.24 (1.05, 1.47)*	1.19 (1.03, 1.38)*
***Potentially confounding variables***		
*Works on a high-risk unit*^b^^f^: No	Referent	Referent
Yes	1.30 (1.15, 1.47)^‡^	1.23 (1.10, 1.37)^‡^
*Age *(in years)^f^	0.99 (0.98, 1.00)^‡^	0.994 (0.992, 0.997)^‡^
*Gender*^f^*: *Female	Referent	Referent
Male	0.82 (0.66, 1.01)	0.87 (0.78, 0.95)*
*Health status, self-reported*^f^		
Poor/fair/good	Referent	Referent
Very good	0.84 (0.73, 0.96)*	0.85 (0.81, 0.89)^‡^
Excellent	0.71 (0.60, 0.84)^‡^	0.71 (0.62, 0.81)^‡^
*Household size* (per person)^f^	1.00 (0.95, 1.04)	NA
*COVID-19 vaccination status*^f^		NA
Incomplete	Referent	
Fully vaccinated	1.78 (1.33, 2.37)	
*Date of submission*^f^		
2021, Jun. 10 to Dec. 31	Referent	Referent
2022, Jan. 1 to Dec. 31	0.94 (0.81, 1.08)	0.94 (0.70, 1.26)
2023, Jan. 1 to Dec. 1	0.47 (0.37, 0.60)^‡^	0.49 (0.32, 0.75)^‡^
*Mitigation*^c^^f^: Low level	Referent	NA
High level	1.26 (0.96, 1.65)	
*Occupation*^f^		
Nurse/NP/midwife	Referent	Referent
Physician/physician assistant	0.65 (0.51, 0.84)^‡^	0.78 (0.70, 0.87)^‡^
Other regulated professional^d^	0.75 (0.64, 0.87)^‡^	0.84 (0.74, 0.95)*
Other^e^	0.85 (0.73, 0.99)*	1.01 (0.87, 1.18)
*Education, highest achieved*^f^		NA
Secondary/College diploma	Referent	
Bachelor’s degree	1.00 (0.87, 1.16)	
Master’s degree	0.88 (0.73, 1.07)	
MD or PhD	0.76 (0.60, 0.95)*	
*Antidepressant, anti-anxiety, or anti-insomnia medication*^f^		NA
Not reported	Referent	
Reported	1.12 (0.98, 1.30)	

*RR* Rate ratio, *NP* Nurse practitioner, *NA* Not applicable

^*^p<0.05

^‡^*p*<0.001

^a^Variance estimates were also adjusted for clustering within province

^b^dult intensive care units, emergency departments, and adult inpatient medical units

^c^Periods of time when multiple COVID-19 transmission mitigation strategies were imposed

^d^Respiratory therapist, laboratory technician, physical therapist, occupational therapist, imaging technician/technologist, pharmacist, pharmacy technician, psychologist, social worker

^e^Infection prevention and control practitioner, food service, ward clerk, administration, healthcare aide, housekeeper, porter, researcher, other clinical support

^f^These variables are included in the adjusted modified Poisson regression model to reduce confounding in the relationship between level of patient contact and dichotomous IES-R scores and should not be interpreted as adjusted main effects, or predictors, in their own right

### Subscale scores

As shown in Table 2, the mean subscale scores were 0.87, 0.87, and 0.67 for avoidance, intrusion, and hyperarousal, respectively. As expected, given that subscale items are summed to create the overall IES-R score, the correlation between the IES-R score and each subscale was 0.92 for both avoidance and hyperarousal and 0.96 for intrusion, with no differences in correlations by year of survey completion. There was also high correlation between the subscale scores at 0.88 for intrusion:hyperarousal, 0.79 for intrusion:avoidance, and 0.74 for hyperarousal:avoidance.

In fully adjusted linear regression models, physical care/contact was associated with increased avoidance and hyperarousal scores, but not intrusion scores. Being in the same room as a patient was also associated with higher avoidance scores.

## Discussion

Provision of physical care during the COVID-19 pandemic was associated with increased concern for PTSD among Canadian HCP. Between June 10, 2021 and December 1, 2023, in adjusted regression models, HCP providing physical care to patients had IES-R scores that were higher than for those with no direct patient contact. Patient contact was also associated with increased avoidance and hyperarousal scores but not with not with intrusion scores.

The association between level of patient contact and emotional distress has been found in previous studies. During the 2003 severe acute respiratory syndrome (SARS) outbreak in Toronto, nurses who had longer contact with patients with SARS had higher distress scores [24]. Similar findings were reported very early in the COVID-19 pandemic (February 2020) in China where working in a frontline position (i.e., directly engaged in clinical activities with patients with elevated temperatures or confirmed to have COVID-19) was associated with significantly higher median IES-R scores (22.5) than those who were not (17.0) [13].

In this study, in 2023, 22.5% of HCP had scores indicative of concern for PTSD. This rate is significantly lower than the 47.8% with scores of concern in 2021 but is, itself, substantially lower than estimates from other studies conducted with HCP that also used the IES-R with a cut-off score of ≥ 24. In a Canadian study, 74% of critical care nurses had scores of concern [25] while in Italy, 65% of physicians and 71% of nurses had scores of concern [26]. However, the estimate from this study is in line with an earlier 2020 Ontario study that also used the IES-R with a cut-off score ≥ 24 that found 50% of HCP had scores suggestive of concern for PTSD [27].

Fattori et al. [28] reported that Italian HCP mean IES-R scores decreased from 22 in 2020/2021 to 13 in 2021/2022. Although the scores are similar to the current study, the decrease in scores occurred about a year earlier in the Italian study. In contrast, Th’ng and colleagues reported substantially lower percentages of emergency department HCP who had scores of concern than in our study, at 13.6–16.2% for 2020 through 2022 in their longitudinal single-centre study in Singapore, with no significant change over time [29].

Studies indicate that HCP have dealt with COVID-19 associated stress in many ways. One in four Canadian HCP reported drinking more in 2021 than before the COVID-19 pandemic [30] and Canadian adults who screened positive for PTSD were 3.5 times more likely than those with a negative screen to report increased cannabis use since the beginning of the pandemic [31]. Conversely, 70% of HCP indicated that they are exercising to improve or maintain their health during the pandemic [30]. Carmassi et al. reported that HCP with IES-R scores of ≥ 24 had significantly higher Work and Social Adjustment Scale [32] scores than HCP with lower scores [33], indicating interference in working, recreational, and social activities, household chores, and family relationships. A rapid review of successful strategies to reduce HCP stress indicated that actions need to be taken at the organizational level [34]. These authors suggest that communication needs to be timely and accurate, and that HCP safety and well-being needs to be kept in the forefront. Healthcare leaders should tailor stress reduction strategies to their population and prioritize HCP who are providing direct patient care.

As with all studies, this study has limitations. Non-random sampling methods were used to generate both the study and the sub-study populations; study participants were self-selected, and some did not complete the IES-R. Biases stemming from differential patterns of study enrollment or attrition could have led to under- or over-estimates of the relationship between level of patient contact and symptoms associated with concern for PTSD. It is unknown if we over- or under-sampled HCP with PTSD symptoms (% of participants with symptoms indicative of concern for PTSD; current study 2021: 47.8%; 2020 Honarmand et al. [27]: 50%; 2021 Crowe et al. [25]: 74%; 2021 Gorini et al.: 65% (physicians) [26]). In addition, the overall variance explained by all regression models was low which is likely due to unmeasured known (e.g., pre-existing mental illness [35], fear of COVID-19 [36], personality [37]), and unknown covariates/confounders/mediators. As well, all results are self-reported and may suffer from social desirability bias. Although we have no data from before (nor early) in the pandemic, one strength of the study is its longitudinal nature, with data from 2021 through 2023. Another strength is that participants were from four provinces across Canada, increasing generalizability.

## Conclusion

HCP who provided direct patient care were significantly more likely to have IES-R scores indicative of concern for PTSD than those reporting no direct patient contact. Healthcare system administrators and planners must consider the high prevalence of concern for PTSD among HCP who worked during the COVID-19 pandemic when reflecting on current human health resources. Early screening for adverse emotional outcomes during stressful times followed by the delivery of timely, tailored preventive strategies is vital for both immediate and long-term HCP health.

### Supplementary Information

Supplementary Material 1

## Data Availability

Study data cannot be shared openly. The datasets generated and/or analyzed during the current study are not publicly available due to information that could compromise the privacy of research participants. Data are available from the corresponding author on reasonable request.

## Abbreviations

PTSD: Post-traumatic stress disorder
HCP: Healthcare provider
IES: Impact of Events Scale
IES-R: Impact of Events Scale-Revised
SARS: Severe acute respiratory syndrome
RR: Rate ratio

## References

[1] WHO Director-General's opening remarks at the media briefing on COVID-19 - 11 March 2020 [press release]. 2020. Accessed 06 May 2024.

[2] Welfare-Wilson A, Adley L, Bell Z, Luby R. COVID-19 and how the wearing of face coverings can affect those with an experience of trauma. J Psychiatr Ment Health Nurs. 2021;28(5):777–82. 33587790 10.1111/jpm.12743 PMC8013392

[3] Bodner E, Bergman YS, Ben-David B, Palgi Y. Vaccination anxiety when vaccinations are available: the role of existential concerns. Stress Health. 2022;38(1):111–8. 34245220 10.1002/smi.3079 PMC8420225

[4] Bryant RA. Post-traumatic stress disorder: a state-of-the-art review of evidence and challenges. World Psychiatry. 2019;18(3):259–69. 31496089 10.1002/wps.20656 PMC6732680

[5] Van Ameringen M, Mancini C, Patterson B, Boyle MH. Post-Traumatic Stress Disorder in Canada. CNS Neurosci Ther. 2008;14(3):171–81. 18801110 10.1111/j.1755-5949.2008.00049.x PMC6494052

[6] Government of Canada. Posttraumatic stress disorder among adults in Canada. Government of Canada; 2024. Available from: https://health-infobase.canada.ca/ptsd-survey/. Accessed 23 Jul 2024.

[7] Ornell F, Halpern SC, Kessler FHP, Narvaez JCM. The impact of the COVID-19 pandemic on the mental health of healthcare professionals. Cad Saude Publica. 2020;36(4):e00063520. 32374807 10.1590/0102-311x00063520

[8] Penninx BWJH, Benros ME, Klein RS, Vinkers CH. How COVID-19 shaped mental health: from infection to pandemic effects. Nat Med. 2022;28(10):2027–37. 36192553 10.1038/s41591-022-02028-2 PMC9711928

[9] Cai Z, Cui Q, Liu Z, Li J, Gong X, Liu J, et al. Nurses endured high risks of psychological problems under the epidemic of COVID-19 in a longitudinal study in Wuhan China. J Psychiatr Res. 2020;131:132–7. 32971356 10.1016/j.jpsychires.2020.09.007 PMC7489269

[10] Crowe S, Howard AF, Vanderspank-Wright B, Gillis P, McLeod F, Penner C, et al. The effect of COVID-19 pandemic on the mental health of Canadian critical care nurses providing patient care during the early phase pandemic: a mixed method study. Intensive Crit Care Nurs. 2021;63:102999. 33342649 10.1016/j.iccn.2020.102999 PMC7832945

[11] Davico C, Ghiggia A, Marcotulli D, Ricci F, Amianto F, Vitiello B. Psychological impact of the COVID-19 pandemic on adults and their children in Italy. Front Psychiatry. 2021;12:572997. 33776812 10.3389/fpsyt.2021.572997 PMC7994767

[12] Kang L, Ma S, Chen M, Yang J, Wang Y, Li R, et al. Impact on mental health and perceptions of psychological care among medical and nursing staff in Wuhan during the 2019 novel coronavirus disease outbreak: a cross-sectional study. Brain Behav Immun. 2020;87:11–7. 32240764 10.1016/j.bbi.2020.03.028 PMC7118532

[13] Lai J, Ma S, Wang Y, Cai Z, Hu J, Wei N, et al. Factors associated with mental health outcomes among health care workers exposed to Coronavirus Disease 2019. JAMA Netw Open. 2020;3(3):e203976. 32202646 10.1001/jamanetworkopen.2020.3976 PMC7090843

[14] Magalhaes E, Stoner A, Palmer J, Schranze R, Grandy S, Amin S, et al. An assessment of mental health outcomes during the COVID-19 pandemic. Community Ment Health J. 2021;57(7):1267–77. 34283359 10.1007/s10597-021-00876-9 PMC8289877

[15] Marcomini I, Agus C, Milani L, Sfogliarini R, Bona A, Castagna M. COVID-19 and post-traumatic stress disorder among nurses: a descriptive cross-sectional study in a COVID hospital. Med Lav. 2021;112(3):241–9. 34142675 10.23749/mdl.v112i3.11129 PMC8223933

[16] Weiss D, Marmar C. The Impact of Events Scale-Revised. In: Wilson J, Keane T, editors. Assessing psychological trauma and PTSD. New York, New York: Guiliford Press; 1996. p. 399–411.

[17] Hong X, Cao J, Wei J, Duan Y, Zhao X, Jiang J, et al. Stress and psychological impact of the COVID-19 outbreak on the healthcare staff at the fever clinic of a tertiary general hospital in Beijing: a cross-sectional study. BJPsych Open. 2021;7(3):e76. 33814026 10.1192/bjo.2021.32 PMC8027548

[18] Horowitz M, Wilner N, Alvarez W. Impact of Events Scale: a measure of subjective stress. Psychosom Med. 1979;41(3):209–18. 472086 10.1097/00006842-197905000-00004

[19] Government of Canada. Summary of evidence supporting COVID-19 public health measures. Government of Canada; 2023. [updated 27 Jan 2023]. Available from: https://www.canada.ca/en/public-health/services/diseases/2019-novel-coronavirus-infection/guidance-documents/summary-evidence-supporting-covid-19-public-health-measures.html. Accessed 23 Jul 2023.

[20] Akanteva A, Dick DW, Amiraslani S, Heffernan JM. Canadian Covid-19 pandemic public health mitigation measures at the province level. Sci Data. 2023;10(1):882-. 38066033 10.1038/s41597-023-02759-y PMC10709578

[21] Zou G. A modified Poisson regression approach to prospective studies with binary data. Am J Epidemiol. 2004;159(7):702–6. 15033648 10.1093/aje/kwh090

[22] Vittinghoff E, Glidden DV, Shiboski SC, McCulloch CE. Regression methods in biostatistics: linear, logistic, survival, and repeated measures models. 1st ed. New York, NY: Springer; 2007.

[23] Government of Canada. FAQ: COVID-19 vaccine. 2022. [updated 09 Dec 2022]. Available from: https://www.canada.ca/en/department-national-defence/campaigns/covid-19/resuming-work/frequently-asked-questions/vaccines-immunization.html. Accessed 08 Aug 2024.

[24] Maunder R. The experience of the 2003 SARS outbreak as a traumatic stress among frontline healthcare workers in Toronto: lessons learned. Philos Trans R Soc Lond B Biol Sci. 2004;359(1447):1117–25. 15306398 10.1098/rstb.2004.1483 PMC1693388

[25] Crowe S, Fuchsia Howard A, Vanderspank B. The mental health impact of the COVID-19 pandemic on Canadian critical care nurses. Intensive Crit Care Nurs. 2022;71:103241-. 10.1016/j.iccn.2022.103241 PMC8919770 35396101

[26] Gorini A, Giuliani M, Fiabane E, Bonomi A, Gabanelli P, Pierobon A, et al. Prevalence of psychopathological symptoms and their determinants in four healthcare workers’ categories during the second year of COVID-19 pandemic. Int J Environ Res Public Health. 2022;19(20):13712. 36294291 10.3390/ijerph192013712 PMC9602535

[27] Honarmand K, Yarnell CJ, Young-Ritchie C, Maunder R, Priestap F, Abdalla M, et al. Personal, professional, and psychological impact of the COVID-19 pandemic on hospital workers: a cross-sectional survey. PLoS One. 2022;17(2):e0263438. 35167590 10.1371/journal.pone.0263438 PMC8846533

[28] Fattori A, Comotti A, Mazzaracca S, Consonni D, Bordini L, Colombo E, et al. Long-term trajectory and risk factors of healthcare workers’ mental health during COVID-19 pandemic: a 24 month longitudinal cohort study. Int J Environ Res Public Health. 2023;20(5):4586. 36901597 10.3390/ijerph20054586 PMC10002366

[29] Th’ng F, Rao KA, Ge L, Neo HN, Molina JAD, Lim WY, et al. Longitudinal study comparing mental health outcomes in frontline emergency department healthcare workers through the different waves of the COVID-19 pandemic. Int J Environ Res Public Health. 2022;19(24):16878. 36554759 10.3390/ijerph192416878 PMC9779183

[30] Statistics Canada. Health care workers’ stress, alcohol consumption and positive health behaviours during the COVID-19 pandemic. 2024. [updated 15 Apr 2024]. Available from: https://www150.statcan.gc.ca/n1/pub/11-627-m/11-627-m2024017-eng.htm. Accessed 24 Apr 2024.

[31] Government of Canada. Cycle 2: Symptoms of posttraumatic stress disorder (PTSD) during the COVID-19 pandemic. Government of Canada; 2022. [updated 19 Oct 2022]. Available from: https://www.canada.ca/en/public-health/services/publications/diseases-conditions/cycle-2-symptoms-posttraumatic-stress-disorder-covid-19-pandemic.html. Accessed 07 May 2024.

[32] Mundt JC, Marks IM, Shear MK, Greist JH. The Work and Social Adjustment Scale: a simple measure of impairment in functioning. Br J Psychiatry. 2002;180:461–4. 11983645 10.1192/bjp.180.5.461

[33] Carmassi C, Pedrinelli V, Dell’Oste V, Bertelloni CA, Grossi C, Gesi C, et al. PTSD and depression in healthcare workers in the Italian epicenter of the COVID-19 outbreak. Clin Pract Epidemiol Ment Health. 2021;17(1):242–52. 35173794 10.2174/1745017902117010242 PMC8728562

[34] Callus E, Bassola B, Fiolo V, Bertoldo EG, Pagliuca S, Lusignani M. Stress reduction techniques for health care providers dealing with severe Coronavirus infections (SARS, MERS, and COVID-19): a rapid review. Front Psychol. 2020;11:589698-. 33362654 10.3389/fpsyg.2020.589698 PMC7758192

[35] Qi G, Yuan P, Qi M, Hu X, Shi S, Shi X. Influencing factors of high PTSD among medical staff during COVID-19: evidences from both meta-analysis and subgroup analysis. Saf Health Work. 2022;13(3):269–78. 35784492 10.1016/j.shaw.2022.06.003 PMC9233879

[36] Lee S, Kim HR, Kim B. The impact of fear of COVID-19 on the impact of event and indirect trauma. Arch Psychiatr Nurs. 2022;41:306–11. 36428065 10.1016/j.apnu.2022.09.003 PMC9458603

[37] Plomecka M, Gobbi S, Neckels R, Radzinski P, Skorko B, Lazzeri S, et al. Factors associated with psychological disturbances during the COVID-19 pandemic: multicountry online study. JMIR Ment Health. 2021;8(8):e28736-e. 34254939 10.2196/28736 PMC8396308

