# Supplementary material for: Level of patient contact and Impact of Event scores among Canadian healthcare providers during the COVID-19 pandemic
Source: BMC Health Serv Res. 2024 Aug 20;24:947. doi: 10.1186/s12913-024-11426-w (PMC11334392; doi:10.1186/s12913-024-11426-w)
Supplement: Supplementary file 1 — Supplementary Material 1 [file 12913_2024_11426_MOESM1_ESM.docx]

**Supplementary Table 1: Linear regression models of IES-R sub-scale scores, Canadian healthcare providers (June 10, 2021-December 1, 2023), N=1498**

| Variable | Adjusted model^1^  Avoidance | Adjusted model^1^  Intrusion | Adjusted model^1^  Hyperarousal |  |
| --- | --- | --- | --- | --- |
| R-squared | 0.082 | 0.084 | 0.092 |  |
| Mean subscale score | 0.87 (0.12, 1.62) | 0.87 (0.25, 1.50) | 0.67 (0.17, 1.33) |  |
| *Level of patient contact*  No direct contact  Never/rarely in room  Same room  Physical care/contact | Referent  0.02 (-0.11, 0.15)  0.08 (0.01, 0.16)*  0.12 (0.01, 0.24)* | Referent  0.01 (-0.06, 0.08)  0.03 (-0.16, 0.23)  0.11 (-0.06, 0.28) | Referent  0.04 (-0.17, 0.25)  0.02 (-0.10, 0.13)  0.09 (0.02, 0.16)* |  |
| ***Potentially confounding variables*** | | | | |
| *Works on a high-risk unit*^2†^: No  Yes | Referent  0.12 (-0.01, 0.25) | Referent  0.17 (-0.01, 0.35) | Referent  0.17 (0.05, 0.29)* |  |
| *Age*^†^ (in years) | -0.007 (-0.014, -0.001)* | -0.002 (-0.007, 0.002) | -0.001 (-0.004, 0.002) |  |
| *Gender*^†^*:* Female  Male | Referent  -0.09 (-0.24, 0.05) | Referent  -0.12 (-0.17, -0.06)* | Referent  -0.16 (-0.24. -0.07)* |  |
| *Health status, self-reported*^†^  Poor/fair/good  Very good  Excellent | Referent  -0.06 (-0.12, 0.01)  -0.24 (-0.43, -0.04)* | Referent  -0.06 (-0.19, 0.06)  -0.23 (-0.42, -0.04)* | Referent  -0.17 (-0.24, -0.10)*  -0.32 (-0.55, -0.10)* |  |
| *Calendar year of submission*^†^  2021  2022  2023 | Referent  0.01 (-0.36, 0.37)  -0.37 (-0.67, -0.06)* | Referent  -0.08 (-0.44, 0.29)  -0.43 (-0.68, -0.17)* | Referent  -0.07 (-0.42, 0.28)  -0.40 (-0.63, -0.17)* |  |
| *Occupation*^†^  Nurse/NP/Midwife  Physician/PA  Other regulated HCP^3^  Other^4^ | Referent  -0.14 (-0.26, -0.02)*  -0.09 (-0.22, 0.04)  -0.03 (-0.13, 0.07) | Referent  -0.19 (-0.39, 0.01)  -0.18 (-0.29, -0.07)*  -0.06 (-0.15, 0.03) | Referent  -0.19 (-0.32, -0.06)*  -0.16 (-0.30, -0.02)*  -0.07 (-0.12, -0.02)* |  |

* p<0.05

HCP: Healthcare provider; NP: nurse practitioner; PA: physician assistant

^1^ Variance estimates were adjusted for clustering within province

^2^ Adult intensive care units, emergency departments, and adult inpatient medical units

^3^ Respiratory therapist, laboratory technician, physical therapist, occupational therapist, imaging technician/technologist, pharmacist, pharmacy technician, psychologist, and social worker

^4^ Infection prevention and control practitioner, food service, ward clerk, administration, healthcare aide, housekeeper, porter, researcher, and other clinical support

^†^ These variables are included in the adjusted modified Poisson regression model to reduce confounding in the relationship between level of patient contact and dichotomous subscale scores and should not be interpreted as adjusted main effects, or predictors, in their own right
